# ﻿A new species of *Amolops* (Amphibia, Anura, Ranidae) from Guizhou Province, China

**DOI:** 10.3897/zookeys.1189.115621

**Published:** 2024-01-12

**Authors:** Shi-Ze Li, Jing Liu, Xiao-Cong Ke, Gang Cheng, Bin Wang

**Affiliations:** 1 Department of Food Science and Engineering, Moutai Institute, Renhuai 564500, China Chinese Academy of Sciences Chengdu China; 2 Chengdu Institute of Biology, Chinese Academy of Sciences, Chengdu 610041, China Moutai Institute Renhuai China; 3 Guizhou Yahua Forestry Engineering Design Consulting Co., Ltd., Guiyang, 550002, China Guizhou Yahua Forestry Engineering Design Consulting Co., Ltd. Guiyang China; 4 College of Materials Science and Engineering, Guiyang College, Guiyang, 550002, China College of Materials Science and Engineering, Guiyang College Guiyang China

**Keywords:** Mitochondrial gene, taxonomy

## Abstract

The Torrent frogs of the genus *Amolops* are widely distributed in Nepal and northern India eastwards to southern China and southwards to Malaysia. The genus currently contains 84 species. Previous studies indicated underestimated species diversity in the genus. In the context, a new species occurring from the mountains in the northwestern Guizhou Province, China is found and described based on morphological comparisons and molecular phylogenetic analyses, *Amolopsdafangensis***sp. nov.** Phylogenetic analyses based on DNA sequences of the mitochondrial 16S rRNA and COI genes supported the new species as an independent lineage. The uncorrected genetic distances between the 16S rRNA and COI genes in the new species and its closest congener were 0.7% and 2.6%, respectively, which are higher than or at the same level as those among many pairs of congeners. Morphologically, the new species can be distinguished from its congeners by a combination of the following characters: body size moderate (SVL 43.2–46.8 mm in males); head length larger than head width slightly; tympanum distinct, oval; vocal sacs absent; vomerine teeth present; dorsolateral folds weak formed by series of glands; nuptial pads present on the base of finger I; heels overlapping when thighs are positioned at right angles to the body; tibiotarsal articulation reaching the level far beyond the tip of the snout when leg stretched forward.

## ﻿Introduction

The Torrent frogs of the genus *Amolops* Cope, 1865 are widespread in Asia, from the southern and eastern Himalayas eastward to the southeastern mainland China and southwards to the Peninsular Malaysia (Wu et al. 2020; Zeng et al. 2020; Frost 2023). The frogs live in the fast-flowing water and occupy specialized features that help them cling to rocks and navigate the turbulent currents (Fei et al. 2009; Fei et al. 2012). The genus currently contains 84 species, of which 51 species have been recorded in China (Fei et al. 2012; Amphibia China 2023; Frost 2023). Recently, according to the phylogenetic framework of the genus, the 84 *Amolops* species were divided into ten species groups, namely the *A.monticola* group, *A.chayuensis* group, *A.hainanensis* group, *A.ricketti* group, *A.spinapectoralis* group, *A.marmoratus* group, *A.larutensis* group, *A.daiyunensis* group, *A.viridimaculatus* group, and the *A.mantzorum* group (Lyu et al. 2019b; Wu et al. 2020; Zeng et al. 2020, 2021; Jiang et al. 2021; Patel et al. 2021; Mahony et al. 2022; Saikia et al. 2022a, 2022b, 2023; Wang et al. 2022; Pham et al. 2023; Qian et al. 2023; Tang et al. 2023; Sheridan et al. 2023). Among them, the *A.mantzorum* group, to which *Amolopsdafangensis* sp. nov. belongs, was proposed by Fei et al. (1999) and is mainly distributed along the eastern margin of the Qinghai-Tibet Plateau (Fei et al. 2009; Lu et al. 2014; Zeng et al. 2020) and currently comprises eleven species (Jiang et al. 2021; Qian et al. 2023; Tang et al. 2023): *Amolopsailao* Tang, Sun, Liu, Luo, Yu & Du, 2023, *A.mantzorum* (David, 1872), *A.granulosus* (Liu & Hu, 1961), *A.loloensis* (Liu, 1950), *A.lifanensis* (Liu, 1945), *A.jinjiangensis* Su, Yang & Li, 1986, *A.tuberodepressus* Liu & Yang, 2000, *A.sangzhiensis* Qian, Xiang, Jiang, Yang & Gui, 2023, *A.shuichengicus* Lyu & Wang, 2019, *A.ottorum* Pham, Sung, Pham, Le, Zieger & Nguyen, 2019, and *A.minutus* Orlov & Ho, 2007. In this species group, *A.ottorum* and *A.minutus* are only known from northwestern Vietnam, and the other species are known from southwestern China (Frost 2023). However, within the group, the phylogenetic relationships between species remain controversial (Lu et al. 2014; Lyu et al. 2019b; Zeng et al. 2020; Wu et al. 2020), and the species diversity of it is also expected to be underestimated (Jiang et al. 2021; Qian et al. 2023; Tang et al. 2023).

Guizhou Province is one of the richest areas for amphibians in China and three *Amolops* species (*A.chaochin*, *A.chunganensis*, and *A.sinensis*) were have been recorded (Amphibia China 2023). During fieldwork in Dafang County, Guizhou Province, some *Amolops* specimens were collected. By our comparisons, these specimens were different from *A.chaochin*, *A.chunganensis*, and *A.sinensis* by the dorsolateral folds being weak, formed by series of glands, and the presence of a circum-marginal groove on the disc of the first finger. Molecular phylogenetic analyses based on mitochondrial DNA and comprehensive morphological comparisons all indicated that the specimens from Dafang County were an undescribed species, herein described as a new species, *Amolopsdafangensis* sp. nov.

## ﻿Materials and methods

### ﻿Sampling

**Figure 1.**
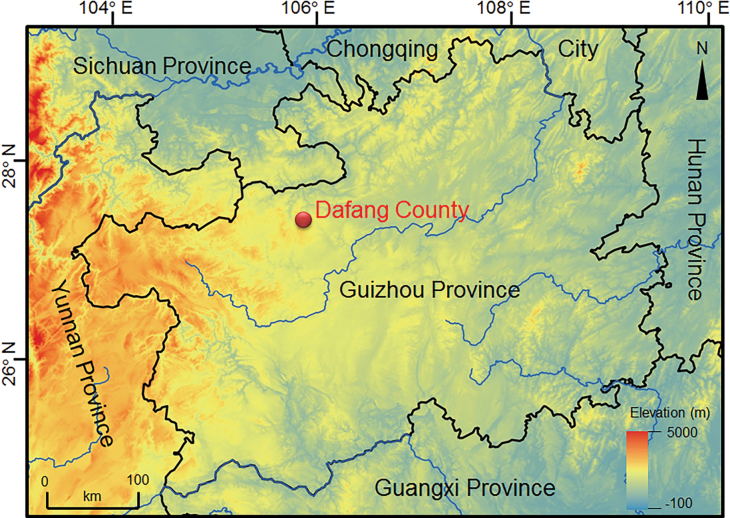
Geographical location of the type locality of *Amolopsdafangensis* sp. nov. in Dafang County, Guizhou Province, China.

Five specimens of *Amolopsdafangensis* sp. nov. including three adult males and two juveniles, were collected from Dafang County, Guizhou Province, China (Fig. 1). All specimens were fixed in 10% buffered formalin for one day, and then transferred to 70% ethanol. Tissue samples were preserved separately in 95% prior to fixation. Specimens collected in this work were all deposited in Maotai Institute (**MT**), Renhuai City, Guizhou Province, China.

### ﻿Collection of molecular data

DNA was extracted from tissue using a standard phenol-chloroform extraction protocol (Sambrook et al. 1989). Two mitochondrial genes, partial 16S ribosomal RNA gene (16S) and cytochrome oxidase subunit I (COI), were amplified. The primers used for 16S were P7 (5’- CGCCTGTTTACCAAAAACAT -3’) and P8 (5’-CCGGTCTGAACTCAGATCACGT’) following Simon et al. (1994), and that for COI were Chmf4 (5’-TYTCWACWAAYCAYAAAGAYATCGG-3’) and Chmr4 (5’-ACYTCRGGRTGRCCRAARAATCA-3’) following Che et al. (2012). PCR amplification reactions were performed in a 30 µl reaction with the following cycling conditions: an initial denaturing step at 95 °C for 4 min; 35 cycles of denaturing at 95 °C for 40 s, annealing at 48 °C/46 °C (16S/COI) for 40 s and extending at 72 °C for 70 s, and a final extending step of 72 °C for 10 min. PCR products were purified with spin columns and then were sequenced with both forward and reverse primers same as PCR. Sequencing was conducted using an ABI Prism 3730 automated DNA sequencer in Chengdu TSING KE Biological Technology Co. Ltd. (Chengdu, China). All sequences were deposited in GenBank (for GenBank Accession numbers refer to Table 1). For phylogenetic analyses, we downloaded corresponding sequences for all related species from GenBank according to previous studies (Qian et al. 2023; Tang et al. 2023; for GenBank accession numbers see Table 1).

**Table 1.** Information for samples used in molecular phylogenetic analyses in this study.

ID	Species	Locality	Voucher number	GenBank accession number	
				16S	COI
1	*Amolopsdafangensis* sp. nov.	Dafang, Guizhou, China	MT DF20230601002	OR936315	OR924345
2	*Amolopsdafangensis* sp. nov.	Dafang, Guizhou, China	MT DF20230601001	OR936314	OR924344
3	*Amolopsdafangensis* sp. nov.	Dafang, Guizhou, China	MT DF20230601003	OR936316	OR924346
4	*Amolopsdafangensis* sp. nov.	Dafang, Guizhou, China	MT DF20230601004	OR936317	OR924347
5	*Amolopsdafangensis* sp. nov.	Dafang, Guizhou, China	MT DF20230601005	OR936318	OR924348
6	*A.mantzorum*	Wolong, Sichuan, China	SCUM 045817HX	MN953706	MN961408
7	*A.mantzorum*	Fengtongzhai, Sichuan, China	SYS a005365	MK573808	MK568323
8	*A.mantzorum*	Dayi, Sichuan, China	SCUM 045825HX	MN953707	MN961409
9	*A.mantzorum*	Mt. Wawu, Sichuan, China	SYS a005337	MK604853	MK605611
10	*A.mantzorum*	Kangding, Sichuan, China	KIZ 041127	MN953764	MN961465
11	*A.mantzorum*	Kangding, Sichuan, China	KIZ 041129	MN953765	MN961466
12	*A.mantzorum*	Fengtongzhai, Sichuan, China	SYS a005366	MK604862	MK605620
13	*A.mantzorum*	Kangding, Sichuan, China	SYS a005356	MK604858	MK605616
14	*A.mantzorum*	Kangding, Sichuan, China	SYS a005357	MK604859	MK605617
15	*A.mantzorum*	Mt. Wawu, Sichuan, China	SYS a005336	MK573804	MK568319
16	*A.ailao*	Mt. Ailao, Xinping, Yunnan, China	GXNU YU000001	MN650752	MN650738
17	*A.ailao*	Mt. Ailao, Xinping, Yunnan, China	GXNU YU000002	MN650753	MN650739
18	*A.tuberodepressus*	Jingdong, Yunnan, China	SCUM 050433CHX	MN953729	MN961432
19	*A.tuberodepressus*	Mt. Wuliang, Yunnan, China	SYS a003931	MK573799	MG991933
20	*A.tuberodepressus*	Jingdong, Yunnan, China	SCUM 050430CHX	MN953730	MN961433
21	*A.tuberodepressus*	Mt. Wuliang, Yunnan, China	SYS a003932	MK573800	MG991934
22	*A.tuberodepressus*	Mt. Ailao, Yunnan, China	SYS a003900	MK573797	MK568314
23	*A.tuberodepressus*	Mt. Ailao, Yunnan, China	SYS a003901	MK573798	MK568315
24	*A.granulosus*	Mt. Guangwu, Sichuan, China	SYS a005399	MK573811	MK568326
25	*A.granulosus*	Mt. Guangwu, Sichuan, China	SYS a005400	MK573812	MK568327
26	*A.granulosus*	Mt. Wawu, Sichuan, China	SYS a005315	MK604850	MK605608
27	*A.granulosus*	Mt. Wawu, Sichuan, China	SYS a005316	MK604851	MK605609
28	*A.granulosus*	China: Dayi, Sichuan	SCUM 045823HX	MN953680	JN700804
29	*A.granulosus*	China: Anxian, Sichuan	SCUM 060911HX	MN953681	MN961381
30	*A.shuichengicus*	Shuicheng, Guizhou, China	SYS a004956	MK604845	MK605603
31	*A.shuichengicus*	Shuicheng, Guizhou, China	SYS a004957	MK604846	MK605604
32	*A.jinjiangensis*	Mt. Gaoligong, Yunnan, China	SYS a004571	MK573801	MK568316
33	*A.jinjiangensis*	Deqing, Yunnan, China	SCUM 050434CHX	MN953700	MN961402
34	*A.jinjiangensis*	Deqing, Yunnan, China	SCUM 050435CHX	EF453741	MN961403
35	*A.jinjiangensis*	Chuxiong, Yunnan, China	KIZ 047905	MN953701	MN961404
36	*A.loloensis*	Zhaojue, Sichuan, China	SYS a005346	MK604854	MK605612
37	*A.loloensis*	Zhaojue, Sichuan, China	SYS a005347	MK604855	MK605613
38	*A.loloensis*	Xichang, Sichuan, China	SCUM 045806HX	MN953704	MN961407
39	*A.loloensis*	Xichang, Sichuan, China	SCUM 045807HX	EF453743	MN961456
40	*A.sangzhiensis*	Mt. Doupeng, Sangzhi, Hunan, China	CSUFT 901	OQ079538	OQ078903
41	*A.sangzhiensis*	Mt. Doupeng, Sangzhi, Hunan, China	CSUFT 907	OQ079540	OQ078905
42	*A.sangzhiensis*	Mt. Doupeng, Sangzhi, Hunan, China	CSUFT 912	OQ079541	OQ078906
43	*A.sangzhiensis*	Mt. Doupeng, Sangzhi, Hunan, China	CSUFT 916	OQ079542	OQ078907
44	*A.sangzhiensis*	Mt. Doupeng, Sangzhi, Hunan, China	CSUFT 927	OQ079543	OQ078908
45	*A.sangzhiensis*	Mt. Doupeng, Sangzhi, Hunan, China	CSUFT 930	OQ079544	OQ078909
46	*A.sangzhiensis*	Mt. Doupeng, Sangzhi, Hunan, China	CSUFT 933	OQ079545	OQ078910
47	*A.lifanensis*	Lixian, Sichuan, China	SYS a005374	MK573809	MK568324
48	*A.lifanensis*	Lixian, Sichuan, China	SYS a005375	MK573810	MK568325
49	*A.lifanensis*	Maoxian, Sichuan, China	SCUM 045801HX	MN953702	MN961405
50	*A.lifanensis*	Maoxian, Sichuan, China	SCUM 045803HX	MN953703	MN961406
51	*A.chunganensis*	Mt. Jinggang, Jiangxi, China	SYS a004212	MK263263	MG991914
52	*A.ricketti*	Mt. Wuyi, Fujian, China	SYS a004141	MK263259	MG991927

### ﻿Phylogenetic analyses and genetic distance

Sequences were assembled and aligned using the Clustalw module in BioEdit 7.0.9.0 (Hall 1999) with default settings. The datasets were checked by eye and revised manually if necessary. Based on the 16S + COI concatenated dataset, phylogenetic analyses were conducted using maximum likelihood (ML) and Bayesian Inference (BI) methods, implemented in PhyML 3.0 (Guindon et al. 2010) and MrBayes 3.12 (Ronquist and Huelsenbeck 2003), respectively. The best-fit model was obtained by the Bayesian inference criteria (BIC) computed with PartitionFinder 2 (Lanfear et al. 2012). In this analysis, 16S gene and each codon position of COI gene were defined, and Bayesian Inference Criteria was used. As a result, the analysis suggested that the best partition scheme is16S gene/each codon position of COI gene, and selected GTR + G + I model as the best model for each partition. For ML analysis, the bootstrap consensus tree inferred from 1000 replicates was used to estimate nodal supports of inferred relationships on phylogenetic trees. For Bayesian analyses, four Markov chains were run for 50 million generations with sampling every 1000 generations. The first 25% of the trees were discarded, representing the burn-in phase of the analyses, and the remaining trees were used to calculate the Bayesian posterior probabilities. Genetic distance between species of *A.mantzorum* group were estimated on 16S and COI genes, respectively, based on uncorrected *p*-distance model using MEGA 6.06 (Tamura et al. 2013).

### ﻿Morphological comparisons

Morphological measurements were made with dial calipers to nearest 0.1 mm by S-ZL following Fei et al. (2009). In total, twenty morphological characteristics were measured for the adult specimens:

**ED** eye diameter (distance from the anterior corner to the posterior corner of the eye);

**FL** foot length (distance from tarsus to the tip of fourth toe);

**HDL** head length (distance from the tip of the snout to the articulation of jaw);

**HDW** maximum head width (greatest width between the left and right articulations of jaw);

**HLL** hindlimb length (maximum length from the vent to the distal tip of the Toe IV);

**IND** internasal distance (minimum distance between the inner margins of the external nares);

**IOD** interorbital distance (minimum distance between the inner edges of the upper eyelids);

**LAL** length of lower arm and hand (distance from the elbow to the distal end of the Finger IV);

**ML** manus length (distance from tip of third digit to proximal edge of inner palmar tubercle);

**NED** nasal to eye distance (distance between the nasal and the anterior corner of the eye);

**NSD** nasal to snout distance (distance between the nasal the posterior edge of the vent);

**LW** lower arm width (maximum width of the lower arm);

**SVL** snout-vent length (distance from the tip of the snout to the posterior edge of the vent);

**SL** snout length (distance from the tip of the snout to the anterior corner of the eye);

**TFL** length of foot and tarsus (distance from the tibiotarsal articulation to the distal end of the Toe IV);

**THL** thigh length (distance from vent to knee);

**TL** tibia length (distance from knee to tarsus);

**TW** maximal tibia width;

**TYD** maximal tympanum diameter;

**UEW** upper eyelid width (greatest width of the upper eyelid margins measured perpendicular to the anterior-posterior axis).

We also compared the morphological characters of the new taxon with other species of *Amolops*. Comparative data were obtained from the literature for all species of *Amolops* (Table 2).

**Table 2.** References for morphological characters for congeners of the genus *Amolops*.

Species	Literature
*A.adicola* Patel, Garg, Das, Stuart & Biju, 2021	Patel et al. 2021
*A.afghanus* (Günther, 1858)	Günther 1858
*A.ailao* Tang, Sun, Liu, Luo, Yu & Du, 2023	Tang et al. 2023
*A.akhaorum* Stuart, Bain, Phimmachak & Spence, 2010	Stuart et al. 2010
*A.albispinus* Sung, Wang & Wang, 2016	Sung et al. 2016
*A.aniqiaoensis* Dong, Rao & Lü, 2005	Zhao et al. 2005
*A.archotaphus* (Inger & Chan-ard, 1997)	Inger and Chan-ard 1997
*A.attiguus* Sheridan, Phimmachak, Sivongxay & Stuart, 2023	Sheridan et al. 2023
*A.assamensis* Sengupta, Hussain, Choudhury, Gogoi, Ahmed & Choudhury, 2008	Sengupta et al. 2008
*A.australis* Chan, Abraham, Grismer & Grismer, 2018	Chan et al. 2018
*A.beibengensis* Jiang, Li, Zou, Yan & Che, 2020	Che et al. 2020
*A.bellulus* Liu, Yang, Ferraris & Matsui, 2000	Liu et al. 2000
*A.binchachaensis* Rao, Hui, Ma & Zhu, 2022“2020”	Zhu and Rao 2022
*A.chakrataensis* Ray, 1992	Ray 1992
*A.chanakya* Saikia, Laskar, Dinesh, Shabnam & Sinha, 2022	Saikia et al. 2022a
*A.chaochin* Jiang, Ren, Lyu & Li, 2021	Jiang et al. 2021
*A.chayuensis* Sun, Luo, Sun & Zhang, 2013	Sun et al. 2013
*A.chunganensis* (Pope, 1929)	Pope 1929
*A.compotrix* (Bain, Stuart & Orlov, 2006)	Bain et al. 2006
*A.cremnobatus* Inger and Kottelat, 1998	Inger and Kottelat 1998
*A.cucae* (Bain, Stuart & Orlov, 2006)	Bain et al. 2006
*A.daiyunensis* (Liu & Hu, 1975)	Liu and Hu 1975
*A.daorum* (Bain, Lathrop, Murphy, Orlov & Ho, 2003)	Bain et al. 2003
*A.deng* Jiang, Wang & Che, 2020	Che et al. 2020
*A.formosus* (Günther, 1876)	Günther 1876 “1875”
*A.gerbillus* (Annandale, 1912)	Annandale 1912
*A.gerutu* Chan, Abraham, Grismer & Grismer, 2018	Chan et al. 2018
*A.granulosus* (Liu & Hu, 1961)	Liu and Hu 1961
*A.hainanensis* (Boulenger, 1900)	Boulenger 1900 “1899”
*A.himalayanus* (Boulenger, 1888)	Boulenger 1888
*A.hongkongensis* (Pope & Romer, 1951)	Pope and Romer 1951
*A.indoburmanensis* Dever, Fuiten, Konu & Wilkinson, 2012	Dever et al. 2012
*A.iriodes* (Bain & Nguyen, 2004)	Bain and Nguyen 2004
*A.jaunsari* Ray, 1992	Ray 1992
*A.jinjiangensis* Su, Yang & Li, 1986	Su et al. 1986
*A.kaulbacki* (Smith, 1940)	Smith 1940
*A.kohimaensis* Biju, Mahony & Kamei, 2010	Biju et al. 2010
*A.kottelati* Sheridan, Phimmachak, Sivongxay & Stuart, 2023	Sheridan et al. 2023
*A.larutensis* (Boulenger, 1899)	Boulenger 1899a
*A.latopalmatus* (Boulenger, 1882)	Boulenger 1882
*A.lifanensis* (Liu, 1945)	Liu 1945
*A.loloensis* (Liu, 1950)	Liu 1950
*A.longimanus* (Andersson, 1939)	Andersson 1939 “1938”
*A.mahabharatensis* Khatiwada, Shu, Wang, Zhao, Xie & Jiang, 2020	Khatiwada et al. 2020
*A.mantzorum* (David, 1872)	David 1872 “1871”
*A.marmoratus* (Blyth, 1855)	Blyth 1855
*A.medogensis* Li & Rao, 2005	Zhao et al. 2005
*A.mengdingensis* Yu, Wu & Yang, 2019	Yu et al. 2019
*A.mengyangensis* Wu & Tian, 1995	Wu and Tian 1995
*A.minutus* Orlov & Ho, 2007	Orlov and Ho 2007
*A.monticola* (Anderson, 1871)	Anderson 1871
*A.nepalicus* Yang, 1991	Yang 1991
*A.nidorbellus* Biju, Mahony & Kamei, 2010	Biju et al. 2010
*A.nyingchiensis* Jiang, Wang, Xie, Jiang & Che, 2016	Jiang et al. 2016
*A.ottorum* Pham, Sung, Pham, Le, Ziegler & Nguyen, 2019	Pham et al. 2019
*A.pallasitatus* Qi, Zhou, Lyu, Lu & Li, 2019	Qi et al. 2019
*A.panhai* Matsui & Nabhitabhata, 2006	Matsui and Nabhitabhata 2006
*A.putaoensis* Gan, Qin, Lwin, Li, Quan, Liu & Yu, 2020	Gan et al. 2020b
*A.ricketti* (Boulenger, 1899)	Boulenger 1899b
*A.sangzhiensis* Qian, Xiang, Jiang, Yang & Gui, 2023	Qian et al. 2023
*A.senchalensis* Chanda, 1987	Chanda 1987
*A.sengae* Sheridan, Phimmachak, Sivongxay & Stuart, 2023	Sheridan et al. 2023
*A.shihaitaoi* Wang, Li, Du, Hou & Yu, 2022	Wang et al. 2022
*A.shuichengicus* Lyu & Wang, 2019	Lyu et al. 2019a
*A.siju* Saikia, Sinha, Shabnam & Dinesh, 2023	Saikia et al. 2023
*A.sinensis* Lyu, Wang & Wang, 2019	Lyu et al. 2019b
*A.spinapectoralis* Inger, Orlov & Darevsky, 1999	Inger et al. 1999
*A.tanfuilianae* Sheridan, Phimmachak, Sivongxay & Stuart, 2023	Sheridan et al. 2023
*A.tawang* Saikia, Laskar, Dinesh, Shabnam & Sinha, 2022	Saikia et al. 2022a
*A.teochew* Zeng, Wang, Lyu & Wang, 2021	Zeng et al. 2021
*A.terraorchis* Saikia, Sinha, Laskar, Shabnam & Dinesh, 2022	Saikia et al. 2022b
*A.tonkinensis* (Ahl, 1927 “1926”)	Ahl 1927 “1926”
*A.torrentis* (Smith, 1923)	Smith 1923
*A.truongi* Pham, Pham, Ngo, Sung, Ziegler & Le, 2023	Pham et al. 2023
*A.tuanjieensis* Gan, Yu & Wu, 2020	Gan et al. 2020a
*A.tuberodepressus* Liu & Yang, 2000	Liu and Yang 2000
*A.viridimaculatus* (Jiang, 1983)	Jiang 1983
*A.vitreus* (Bain, Stuart & Orlov, 2006)	Bain et al. 2006
*A.wangyali* Mahony, Nidup, Streicher, Teeling & Kamei, 2022	Mahony et al. 2022
*A.wangyufani* Jiang, 2020	Che et al. 2020
*A.wenshanensis* Yuan, Jin, Li, Stuart & Wu, 2018	Yuan et al. 2018
*A.wuyiensis* (Liu & Hu, 1975)	Liu and Hu 1975
*A.yatseni* Lyu, Wang & Wang, 2019	Lyu et al. 2019
*A.yunkaiensis* Lyu, Wang, Liu, Zeng & Wang, 2018	Lyu et al. 2018

## ﻿Results

### ﻿Phylogenetic analyses

**Figure 2.**
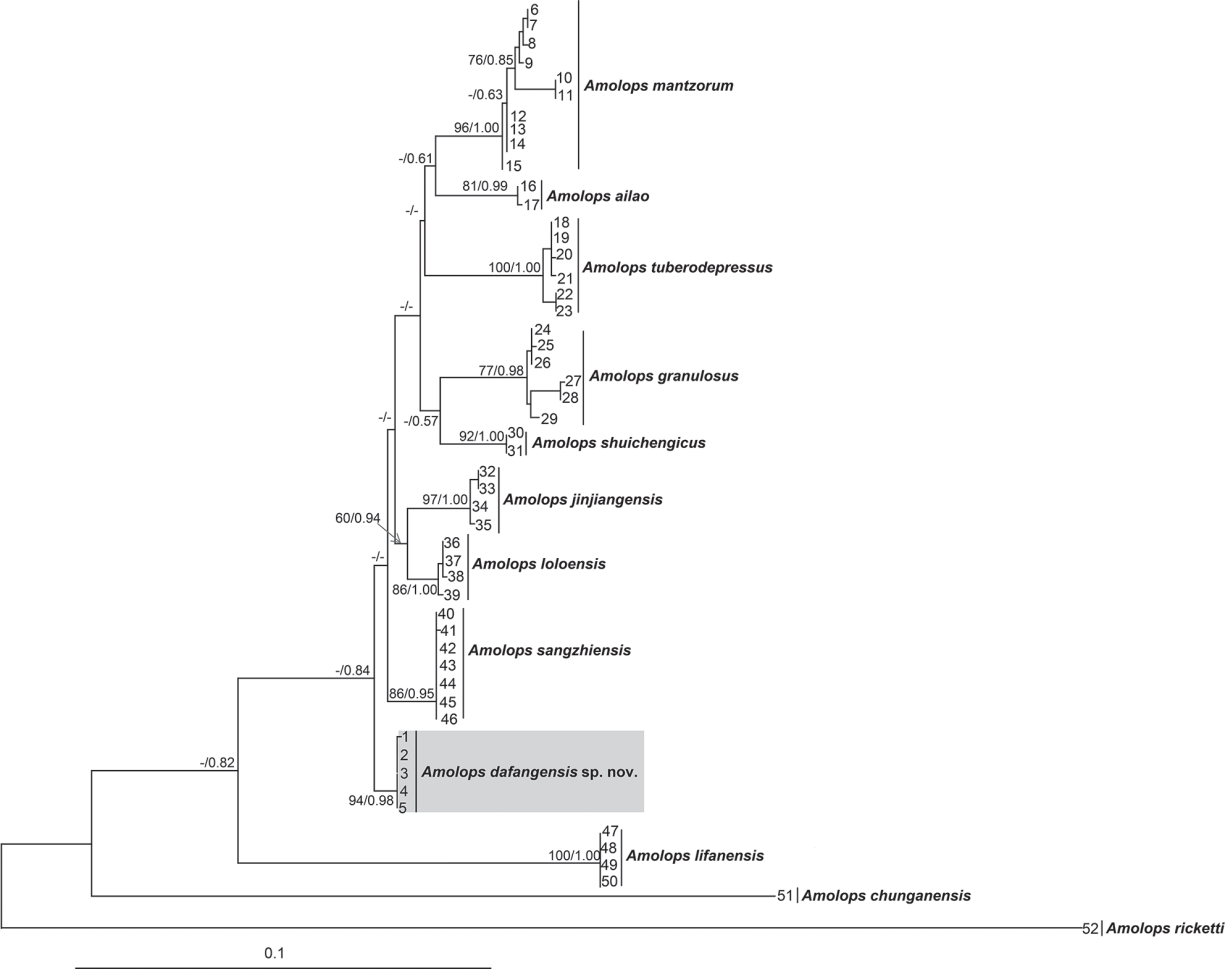
Maximum likelihood (ML) tree of *Amolopsmantzorum* group based on the 16S and CO1 genes. ML bootstrap supports (BS) /Bayesian posterior probability (BPP) were denoted beside each node, and “-” denotes BS < 50% or BPP < 0.60. Samples 1–52 refer to Table 1.

The ML and BI phylogenetic trees were constructed based on concatenated DNA sequences of the mitochondrial 16S (425 bp) and COI (606 bp) genes. ML and BI analyses resulted in essentially identical topologies though some basal relationships between clades were not resolved (Fig. 2). The new taxon was indicated as an independent clade. Furthermore, the smallest uncorrected *p*-distance between *Amolopsdafangensis* sp. nov. and its most closely-related congeners is 0.7% (vs *A.sangzhiensis*) on 16S gene (Suppl. material 1), and 2.6% (vs *A.loloensis*) on COI gene (Suppl. material 2), which was higher or at the same level with those among many pairs of congeners, for example, 0.3% between *A.sangzhiensis* and *A.jinjiangensis* on the 16S gene, and 3.2% between *A.jinjiangensis* and *A.loloensis* on the COI gene.

### ﻿Morphological comparisons

Morphological measurements are given in Table 3. The new taxon could be identified from its congeners by a series of differences in morphological characters.

**Table 3.** Measurements of the adult specimens of *Amolopsdafangensis* sp. nov. Units are given in mm. See abbreviations for the morphological characters in Materials and methods section.

Voucher	MT DF20230601001	MT DF20230601002	MT DF20230601003	Range	Mean ± SD
Sex	male	male	male		
SVL	43.2	44.7	46.8	43.2–46.8	44.9 ± 1.8
HDL	14.5	15.0	15.6	14.5–15.6	14.9 ± 0.6
HDW	14.3	14.7	15.1	14.3–15.1	14.8 ± 0.4
SL	6.1	6.1	6.6	6.1–6.6	6.3 ± 0.3
ED	3.9	4.5	4.3	3.9–4.5	4.3 ± 0.3
UEW	3.5	3.9	3.8	3.5–3.9	3.7 ± 0.2
IOD	4.4	4.1	4.7	4.1–4.7	4.4 ± 0.3
IND	5.2	5.4	5.7	5.2–5.7	5.4 ± 0.3
NED	2.7	2.4	3.0	2.4–3.0	2.7 ± 0.3
NSD	3.2	2.4	3.0	2.4–3.2	2.9 ± 0.4
TYD	1.9	2.4	1.7	1.7–2.4	2.0 ± 0.4
LAL	22.5	24.0	23.5	22.5–24.0	23.3 ± 0.8
LW	3.2	3.8	3.8	3.2–3.8	3.6 ± 0.3
ML	13.8	14.4	14.6	13.8–14.6	14.3 ± 0.4
HLL	80.4	83.4	87.3	80.4–87.3	83.7 ± 3.4
THL	22.3	24.0	24.9	22.3–24.9	23.7 ± 1.3
TL	25.8	26.2	27.9	25.8–27.9	26.6 ± 1.1
TW	5.0	5.4	5.8	5.0–5.8	5.4 ± 0.4
TFL	36.3	38.1	39.5	36.3–39.5	38.0 ± 1.6
FL	22.3	22.8	24.6	22.3–24.6	23.2 ± 1.2

### ﻿Taxonomic account

#### ﻿ Amolops dafangensis sp. nov.

Taxon classificationAnimaliaAnuraRanidae

453188E7-1FA1-5CC8-8E9F-57B3A868D44E

https://zoobank.org/22D19386-8779-4FBC-8BF9-71FB7070403B

Figs 3 , 4 , 5

##### Material examined.

***Holotype*.**MT DF20230601002, adult male, collected by Shize Li on 1 June 2023 in Dafang County (27.40078312°N, 105.92804027°E; elevation 1300 m a.s.l.), Guizhou Province, China. ***Paratypes*.** One male MT DF20230601003 collected by Jing Liu on 1 June 2023, one male MT DF20230601001 and two juveniles MT DF20230601004 and MT DF20230601005 were collected by Xiaocong Ke on 1 June 2023 from the same place as holotype.

##### Diagnosis.

*Amolopsdafangensis* sp. nov. resembles members of the *A.mantzorum* group in the absence of true dorsolateral folds and the presence of a circum-marginal groove on the disc of the first finger. The tarsal fold and tarsal glands are absent, and a nuptial pad is present on the first finger in males (Jiang et al. 2021).

*Amolopsdafangensis* sp. nov. can be distinguished from other congeners by the following characters: (1) body size moderate (SVL 43.2 – 46.8 mm in males); (2) head length larger than head width slightly; (3) tympanum distinct, oval; (4) vocal sacs absent; (5) vomerine teeth present; (6) dorsolateral folds weak formed by series of glands; (7) nuptial pads present on base of finger I; (8) heels overlapping when thighs are positioned at right angles to the body; tibiotarsal articulation reaching the level far beyond the tip of the snout when leg stretched forward.

##### Description of holotype.

**Figure 3.**
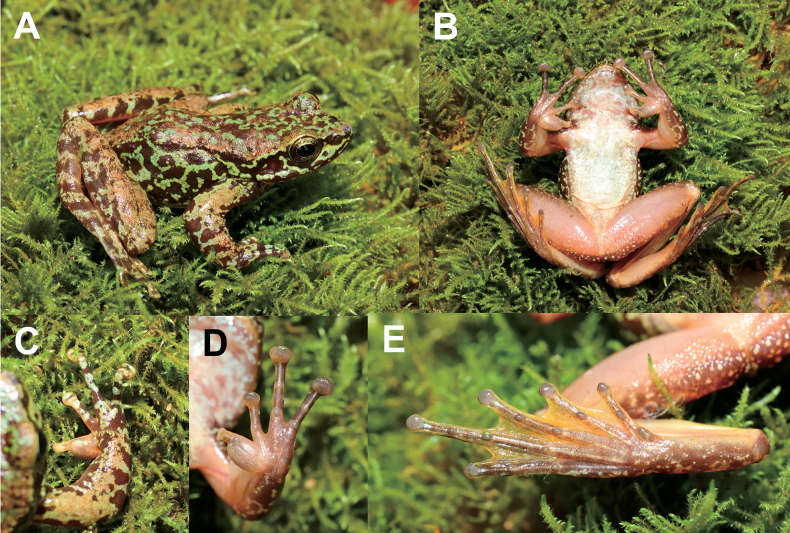
Photographs of the holotype MT DF20230601002 of *Amolopsdafangensis* sp. nov. in life **A** dorsal view **B** ventral view **C** dorsal view of hand **D** ventral view of hand **E** ventral view of foot.

**Figure 4.**
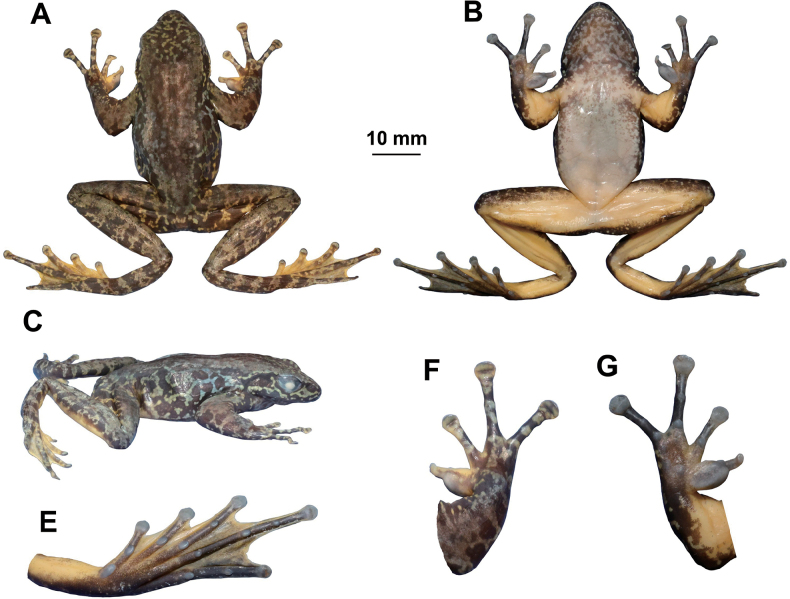
The holotype specimen MT DF20230601002 of *Amolopsdafangensis* sp. nov. **A** dorsal view **B** ventral view **C** lateral view **D** dorsal view of hand **E** ventral view of hand **F** ventral view of foot.

**Adult male** (Figs 3, 4), body size moderate, SVL 44.7 mm. head length larger than head width slightly (HDL: HDW = 1.02); snout short, rounded in dorsal view, projecting beyond lower jaw; eye large and convex, eye diameter 0.74× of snout length; nostril rounded, between to tip of snout and eyes; internasal distance larger than interorbital distance; tympanum circular, distinct, 0.56× of eye diameter; loreal region slightly concave; nares oval; pineal ocellus visible; supratympanic fold extends from back of eye to above shoulder; vomerine teeth present; tongue deeply notched posteriorly; vocal sac absent.

Forelimbs robust (LW/SVL=0.08); lower arm and hand beyond one-second of body length (LAL/SVL=0.51); fingers slender, relative finger lengths I < II < IV < III; finger tips on II–IV dilated to wide cordiform disks with circum-marginal grooves, tip of first finger with small disk but without circum-marginal groove; all fingers without webbing and lateral fringes; subarticular tubercle prominent; supernumerary tubercle indistinct; inner metacarpal tubercle oval, elongate; outer metacarpal tubercles small round; velvety nuptial pad on finger I.

Hindlimbs long, nearly 2× SVL (HLL/SVL = 1.87); tibiotarsal articulation reaching the level far beyond the tip of the snout when leg stretched forward; tibias longer than thigh length, heels overlapped; toes slender, relative lengths I < II < III < V < IV; toes entirely webbed; tips of toes expanded into disc with circum-marginal grooves; outer metatarsal tubercle absent; inner metatarsal tubercle small but well developed.

Skin on dorsum and dorsal surfaces of limbs smooth; dorsolateral folds weak, formed by series of glands been an incomplete line, extending from above shoulder to vent; weak dorsolateral glandular lines; ventral surface of bell and limbs smooth except a few small tubercles on posterior surface of thigh and around vent.

##### Coloration in life.

In life, iris pale brown with dark wash; top of head and dorsum golden brown with large rounded black brown and green spots; sides of head with a pale green stripe extending from loreal region to region behind and below eye along upper lip; a black brown band from the tip of the snout through the nostril to an anterior border of the eye, continuing behind the eye to the shoulder; temporal region black brown with green blotches; the flank green with some back brown spots; limbs dorsally golden brown with black brown bands; chest and venter white, throat white with pale brown; ventral surface of anterior forelimbs brown with green spots; finger I and II fresh-colored, finger III and IV brown; ventral surface of hindlimbs fresh-colored (Fig. 3).

##### Color in preservative.

Dorsal surface fade to pale brown with beige brown and black spots on head, flank and on limbs; ventral surface fade to creamy white, marbled with brown on throat and chest (Fig. 4).

##### Variation.

**Figure 5.**
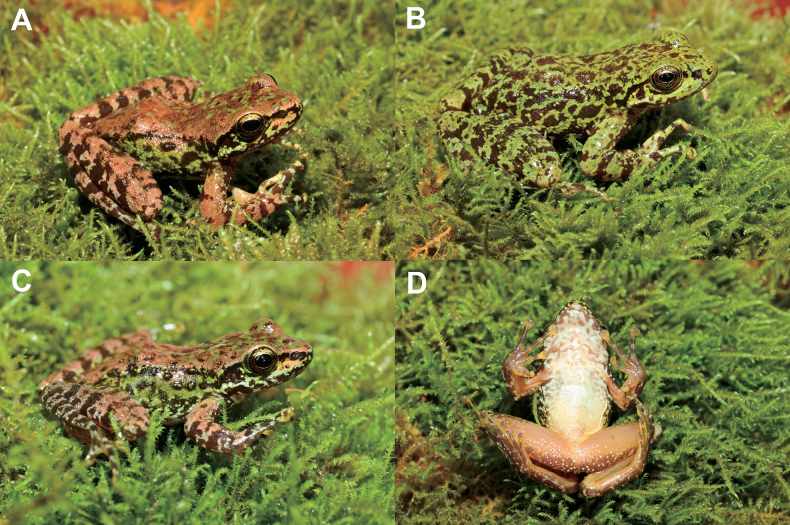
Color variation in *Amolopsdafangensis* sp. nov. **A** dorsolateral view of the male specimen MT DF20230601001 **B** dorsolateral view of the male specimen MT DF20230601003 **C** dorsolateral view of the juvenile specimen MT DF20230601004 **D** ventral view of the male specimen juvenile specimen MT DF20230601004

Measurements of all specimens are listed in Table 3. All specimens were very similar in morphology, but in MT DF20230601001 the dorsum was golden brown with few green spots (Fig. 5A); in MT DF20230601003 the dorsum and dorsal surfaces of limbs were green with brown spots (Fig. 5B); in the juvenile specimen MT DF20230601004 the flank was mainly green with black spots and the ventral surface of the throat and chest were white with pale brown spots (Fig. 5C, D).

##### Secondary sexual characteristics.

Adult males lack vocal sacs. In breeding, pale yellow glandular nuptial pads are present on finger I in males.

##### Morphological comparisons.

The molecular phylogenetic results placed the new species as an independent clade into *A.marmoratus* group. Within the *A.mantzorum* group, the new species can be distinguished from *A.ailao* by having a larger body size (adult males SVL 43.2–46.8 mm vs 33.0–35.1 mm); by vomerine teeth present (vs absent), and by tibiotarsal articulation reaching the level far beyond the tip of the snout when leg stretched forward (vs reaching beyond anterior corner of eye); differs from *A.granulosus* by having a smooth dorsum skin (vs rough with spinules in males) and the absence of vocal sacs in males (vs present); differs from *A.lifanensis* by having a smaller body size (adult males SVL 43.2–46.8 mm vs 52.0–56.0) and having distinct tympanum (vs indistinct); differs from *A.mantzorum* by having a smaller body size (adult males SVL 43.2 – 46.8 mm vs 49.0–57.0 mm), head length about equal to or larger than head width (vs head length smaller than head width); differs from *A.minutus* by having a larger body size (adult males SVL 43.2–46.8 mm vs 29.70–36.42 mm), and the absence of vocal sacs and gular pouches in males (vs well developed); differs from *A.ottorum* by the presence of vomerine teeth (vs absent); differs from *A.shuichengicus* by having a larger body size in males (adult males SVL 43.2–46.8 mm vs 34.6–39.6 mm), and having weak dorsolateral glandular lines (vs strong dorsolateral folds); differs from *A.tuberodepressus* by having a smaller body size (adult males SVL 43.2–46.8 mm vs 48–56mm), and by having weak dorsolateral glandular lines (vs absent); differs from *A.jinjiangensis* by having distinct tympanum (vs indistinct).

*Amolopsdafangensis* sp. nov. is phylogenetically most closed to *A.loloensis* and *A.sangzhiensis*, and the new species could be distinguished from *A.loloensis* by having a smaller body size in males (adult males SVL 43.2–46.8 mm vs 55–62 mm), having distinct tympanum (vs indistinct), tibiotarsal articulation reaching the level far beyond the tip of the snout when leg stretched forward (vs just reaching eye or nostrils), spots on head and dorsum irregular (vs spots on head and dorsum round or oval); differs from *A.sangzhiensis* by having a larger body size in males (adult males SVL 43.2–46.8 mm vs 40.3–40.9 mm), having distinct tympanum (vs indistinct),tibiotarsal articulation reaching the level far beyond the tip of the snout when leg stretched forward (vs just reaching nostrils), mouth corner smooth (vs with dense spiny tubercles around the mouth corner).

*Amolopsdafangensis* sp. nov. differs from the species of the *A.monticola* group namely *A.adicola*, *A.akhaorum*, *A.aniqiaoensis*, *A.archotaphus*, *A.bellulus*, *A.binchachaensis*, *chakrataensis*, *A.chaochin*, *A.chunganensis*, *A.compotrix*, *A.cucae*, *A.daorum*, *A.deng*, *A.iri*, *A.kohimaensis*, *A.mengdingensis*, *A.mengyangensis*, *A.monticola*, *A.nyingchiensis*, *A.putaoensis*, *A.truongi*, *A.tuanjieensis*, *A.vitreus*, and *A.wenshanensis* by dorsolateral folds weak formed by series of glands (vs truth dorsolateral folds present), further distinguished from *A.adicola*, *A.akhaorum*, *A.aniqiaoensis*, *A.archotaphus*, *A.chaochin*, *A.chunganensis*, *A.compotrix*, *A.cucae*, *A.daorum*, *A.iriodes*, *A.kohimaensis*, *A.mengdingensis*, *A.mengyangensis*, *A.monticola*, *A.putaoensis*, *A.truongi*, *A.tuanjieensis*, *A.vitreus*, and *A.wenshanensis* by vocal sac absent (vs present).

*Amolopsdafangensis* sp. nov. differs from *A.chayuensis*, the sole member of the *A.chayuensis* group, by dorsolateral folds weak formed by series of glands (vs truth dorsolateral folds present), and vocal sacs absent (vs present).

*Amolopsdafangensis* sp. nov. differs from the *A.viridimaculatus* group contains 14 species, namely *A.beibengensis*, *A.chanakya*, *A.formosus*, *A.himalayanus*, *A.kaulbacki*, *A.longimanus*, *A.medogensis*, *A.nidorbellus*, *A.pallasitatus*, *A.senchalensis*, *A.tawang*, *A.wangyali*, *A.wangyufani*, and *A.viridimaculatus* by dorsolateral folds weak formed by series of glands (vs dorsolateral folds absent) and smaller body size (vs male SVL 75.8 mm in *A.beibengensis*, male SVL 76.4 mm in *A.chanakya*, males SVL 61.3–63.1 mm in *A.formosus*, male SVL 80 mm in *A.himalayanus*, males SVL 70–72 mm in *A.kaulbacki*, male SVL 95 mm in *A.medogensis*, males SVL 76.4–82.3 mm in *A.nidorbellus*, male SVL 46.2 mm in *A.senchalensis*, male SVL 82.5 mm in *A.tawang*, males SVL 71.4–76.7 mm in *A.wangyali*, males SVL 68.3–69.0 mm in *A.wangyufani*, and males SVL 72.7–82.3 mm in *A.viridimaculatus*).

*Amolopsdafangensis* sp. nov. differs from the *A.marmoratus* group of 13 species (*A.afghanus*, *A.assamensis*, *A.gerbillus*, *A.indoburmanensis*, *A.jaunsari*, *A.latopalmatus*, *A.mahabharatensis*, *A.marmoratus*, *A.nepalicus*, *A.panhai*, *A.siju*, and *A.terraorchis*) by circum-marginal groove on disc of finger I absent (vs present), and vocal sac absent (vs present with the exception of *A.siju*).

*Amolopsdafangensis* sp. nov. differs from *A.spinapectoralis*, the sole member of the *A.spinapectoralis* group, by circum-marginal groove on disc of finger I absent (vs present), and vocal sac absent (vs present).

*Amolopsdafangensis* sp. nov. differs from the *A.larutensis* group with eight species, namely *A.attiguus*, *A.australis*, *A.cremnobatus*, *A.gerutu*, *A.kottelati*, *A.larutensis*, *A.sengae*, and *A.tanfuilianae* by circum-marginal groove on disc of finger I absent (vs present), and vocal sac absent (vs present).

*Amolopsdafangensis* sp. nov. differs from the *A.ricketti* group that contains eight species (*A.shihaitaoi*, *A.sinensis*, *A.ricketti*, *A.wuyiensis*, *A.yunkaiensis*, *A.albispinus*, *A.yatseni*, and *A.tonkinensis*) by circum-marginal groove on disc of finger I absent (vs present), dorsolateral glandular folds present (vs absent), and nuptial pad without conical or papillate nuptial spines (vs present).

*Amolopsdafangensis* sp. nov. differs from the *A.daiyunensis* group of three species, namely *A.daiyunensis*, *A.teochewiensis* and *A.teochew*, by circum-marginal groove on disc of finger I absent (vs present), vomerine teeth present (vs absent) and and vocal sac absent (vs present).

*Amolopsdafangensis* sp. nov. differs from the *A.hainanensis* group (*A.hainanensis* and *A.torrentis*) by vomerine teeth present (vs absent) and further differs from *A.hainanensis* by having a smaller body size (adult males SVL 43.2–46.8 mm vs 71–93 mm) and circum-marginal groove on disc of finger I absent (vs present); further differs from *A.torrentis* by having a larger body size (adult males SVL 43.2–46.8 mm vs 28–33 mm) and vocal sac absent (vs present).

##### Distribution and ecology.

**Figure 6.**
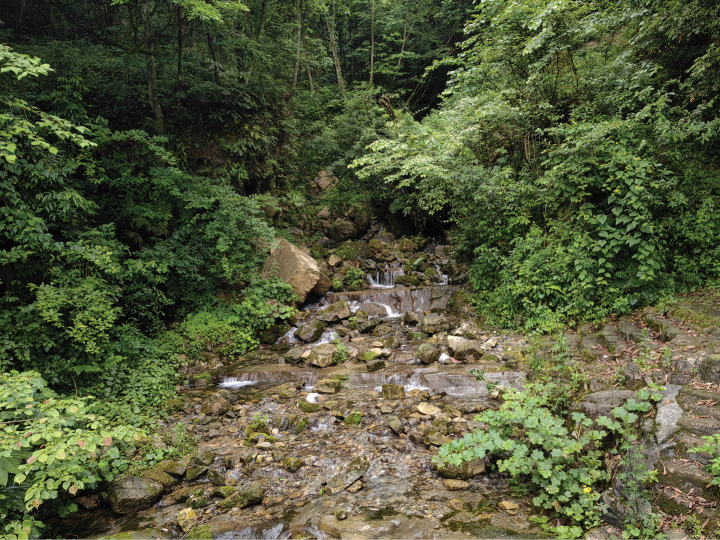
Habitat of *Amolopsdafangensis* sp. nov. in the type locality, Dafang County, Guizhou Province, China.

At present, *Amolopsdafangensis* sp. nov. was only found on vegetation in a mountain stream in Dafang County, Guizhou Province, China at approximately 1600 m elevation. The rocks of this stream are covered with moss, and low vegetation grows out of the cracks (Fig. 6). We did not find eggs, nor *Amolopsdafangensis* sp. nov. tadpoles or females, and advertisement calls were not recorded, but we observed distinct nuptial pad in the males. Based on our surveys, we speculate that the breeding season is probably in early June. *Boulenophrysjiangi* (Liu, Li, Wei, Xu, Cheng, Wang & Wu, 2020), *Boulenophrysqianbeiensis* (Su, Shi, Wu, Li, Yao, Wang & Li, 2020), and *Leptobrachellajinshaensis* Cheng, Shi, Li, Liu, Li & Wang, 2021were also found in the type locality.

##### Etymology.

The specific epithet *dafangensis* refers to the distribution of this species, Dafang County, Guizhou Province, China. We propose the common English name “Dafang cascade frogs” for this species and Chinese name as “Da Fang Tuan Wa (大方湍蛙)”.

## ﻿Discussion

In this study, we describe a new species based on morphological comparisons and molecular phylogenetic analyses; although the genetic distance between the new species and its most closely-related congeners is 0.7% for the 16S gene, the morphological characters differ from those of other species of the genus *Amolops*. This small genetic difference is likely due to the limited phylogenetic information content in this particular gene fragment (Chan et al. 2022). Speciation usually begins with spatial isolation or adaptation to unique environments without strict isolation (Schilthuizen 2000). Significant spatial isolation and subsequent formation of unique lineages may be due to isolation or long-range dispersal across barriers such as mountains, rivers, or other intervening unsuitable habitats (Mayr 1963; Avise 2000; Rundle and Nosil 2005; Schluter 2009). The geographical distances between *Amolopsdafangensis* sp. nov. and its closely-related congeners *A.sangzhiensis* and *A.loloensis* are more than 800 km and 370 km, respectively, and the type locality of the three species are in different mountains: the new species is distributed in Dalou Mountains, *A.sangzhiensis* in easternmost Wuling Mountains, and *A.loloensis* in the Daliangshan Mountains, with significantly different biota. Therefore, we speculate that isolation is likely to have promoted speciation between the lineages and led to the evolution of different morphologies between the new species, *A.sangzhiensis*, and *A.loloensis*.

In the last five years, 25 new frog species have been described in Guizhou Province, China (Frost 2023). Dafang County is in the northwest of Guizhou Province, China, and there have been few surveys of amphibians in the area over the years. From 2020 to 2023 we conducted five surveys in this region. Only in June 2023 was the new species discovered, and only three adult males and two juveniles were found in a range of ~ 100 meters below the source of the stream. Therefore, we infer that the population of the new species is small. We recommend the new species be assigned as vulnerable (VU) according to the evaluation criteria of the IUCN Red List of threatened Species (IUCN 2012). Future research should focus on determining the distribution and elevational range of the species.

## Supplementary Material

XML Treatment for Amolops dafangensis

## ﻿Additional information

### Author ORCIDs

Bin Wang https://orcid.org/0000-0001-6036-5579

### Data availability

All of the data that support the findings of this study are available in the main text or Supplementary Information.

## Supplementary materials

Supplementary material 1 Uncorrected p-distances between species in the *Amolopsmantzorum* group based on the 16S gene sequences Data type xlsx https://binary.pensoft.net/file/966439This dataset is made available under the Open Database License (http://opendatacommons.org/licenses/odbl/1.0/). The Open Database License (ODbL) is a license agreement intended to allow users to freely share, modify, and use this Dataset while maintaining this same freedom for others, provided that the original source and author(s) are credited.Shi-Ze Li, Jing Liu, Xiao-Cong Ke, Gang Cheng, Bin Wang

Supplementary material 2 Uncorrected p-distances between species in the *Amolopsmantzorum* group based on the COI gene sequences Data type xlsx https://binary.pensoft.net/file/966440This dataset is made available under the Open Database License (http://opendatacommons.org/licenses/odbl/1.0/). The Open Database License (ODbL) is a license agreement intended to allow users to freely share, modify, and use this Dataset while maintaining this same freedom for others, provided that the original source and author(s) are credited.Shi-Ze Li, Jing Liu, Xiao-Cong Ke, Gang Cheng, Bin Wang
